# Adult Expression of Tbr2 Is Required for the Maintenance but Not Survival of Intrinsically Photosensitive Retinal Ganglion Cells

**DOI:** 10.3389/fncel.2022.826590

**Published:** 2022-03-23

**Authors:** Sadaf Abed, Andreea Reilly, Sebastian J. Arnold, David A. Feldheim

**Affiliations:** ^1^Department of Molecular, Cell, and Developmental Biology, University of California, Santa Cruz, Santa Cruz, CA, United States; ^2^Institute of Experimental and Clinical Pharmacology and Toxicology, Faculty of Medicine, University of Freiburg, Freiburg, Germany

**Keywords:** melanopsin, EOMES, transcription factor, cell maintenance, optic nerve crush

## Abstract

Retinal ganglion cells expressing the photopigment melanopsin are intrinsically photosensitive (ipRGCs). ipRGCs regulate subconscious non-image-forming behaviors such as circadian rhythms, pupil dilation, and light-mediated mood. Previously, we and others showed that the transcription factor Tbr2 (EOMES) is required during retinal development for the formation of ipRGCs. Tbr2 is also expressed in the adult retina leading to the hypothesis that it plays a role in adult ipRGC function. To test this, we removed *Tbr2* in adult mice. We found that this results in the loss of melanopsin expression in ipRGCs but does not lead to cell death or morphological changes to their dendritic or axonal termination patterns. Additionally, we found ectopic expression of Tbr2 in conventional RGCs does not induce melanopsin expression but can increase melanopsin expression in existing ipRGCs. An interesting feature of ipRGCs is their superior survival relative to conventional RGCs after an optic nerve injury. We find that loss of Tbr2 decreases the survival rate of ipRGCs after optic nerve damage suggesting that *Tbr2* plays a role in ipRGC survival after injury. Lastly, we show that the GABAergic amacrine cell marker Meis2, is expressed in the majority of Tbr2-expressing displaced amacrine cells as well as in a subset of Tbr2-expressing RGCs. These findings demonstrate that *Tbr2* is necessary but not sufficient for melanopsin expression, that *Tbr2* is involved in ipRGC survival after optic nerve injury, and identify a marker for Tbr2-expressing displaced amacrine cells.

## Introduction

The retina comprises six neuronal cell types, each with distinct roles in visual scene detection and processing. Among these are retinal ganglion cells (RGCs) which send axons to > 50 retinorecipient brain regions (Martersteck et al., 2017), and amacrine cells (ACs) which modulate RGC activity. RGCs and ACs can be further divided into > 30 and > 40 subtypes, respectively, based on molecular, morphological, and physiological features (MacNeil and Masland, 1998; Lin and Masland, 2006; Sanes and Masland, 2015; Baden et al., 2016; Bae et al., 2018; Rheaume et al., 2018; Yan et al., 2020). The processes of generating and maintaining this diversity largely remain elusive, but transcription factor codes have been shown to be important for neuronal subtype specification and maintenance (Guillemot, 2007; Deneris and Hobert, 2014; Peng et al., 2017; Sajgo et al., 2017; Lyu et al., 2021).

Neuron types are defined in part by the expression of the genes that contribute to their identity and function, including those that encode sensory receptors, signaling molecules, ion channels, and structural features such as dendritic arborization (Deneris and Hobert, 2014). Our lab and others have shown that the t-box transcription factor, Tbr2 (also known as EOMES), is expressed in a subset of RGCs early in development that will become ipRGCs (defined by expression of melanopsin, and axonal targeting patterns; Mao et al., 2014; Sweeney et al., 2014). Tbr2 is also expressed in a subset of displaced ACs. Removal of Tbr2 from RGCs during development leads to a loss of ipRGCs (Mao et al., 2014; Sweeney et al., 2014). Tbr2 expression is maintained in the adult and is expressed in all ipRGC subtypes (Tran et al., 2019; Chen et al., 2021).

ipRGCs are intrinsically photosensitive because they express the photopigment melanopsin which allows them to detect light and thus execute several light-induced behaviors (Provencio et al., 2000, 2002a; Berson et al., 2002; Hattar et al., 2002), including: Circadian photoentrainment, pupillary light reflex, mood regulation, and learning; ipRGCs also play a role in some aspects of image-forming vision including contrast detection (Panda et al., 2002; Ruby et al., 2002; Güler et al., 2008; Hatori et al., 2008; Legates et al., 2012; Schmidt et al., 2014; Sonoda et al., 2018; Stabio et al., 2018). ipRGCs integrate rod, cone, and melanopsin signals before transmitting this information to many subcortical areas of the brain. Loss of ipRGCs in blinding diseases in humans results in sleep disorders, depression, anxiety, defects in post-illumination pupil response, and loss of light-induced suppression of melatonin secretion (Pérez-Rico et al., 2010; Feigl et al., 2011; Agorastos et al., 2013; Wang et al., 2013; Gracitelli et al., 2016). In mice, loss of ipRGCs results in defects in circadian photoentrainment, the pupillary light reflex, light-suppression of locomotor activity, mood, and learning (Güler et al., 2008; Hatori et al., 2008; Legates et al., 2012).

It has been well-documented that ipRGCs survive after injuries to the optic nerve, however, the reason for their survival is not well understood (Robinson and Madison, 2004; Li et al., 2008; Pérez De Sevilla Müller et al., 2014; Duan et al., 2015). Single-cell RNA-sequencing of RGCs after nerve crush shows that Tbr2 is enriched in the RGCs that survive (Tran et al., 2019), suggesting a role for Tbr2 in RGC survival after injury.

Here, we set out to determine the role that Tbr2 plays in ipRGC maintenance and in ipRGC survival after injury. We employed a tamoxifen-inducible Cre recombinase system to specifically remove Tbr2 during adulthood in cells that endogenously express Tbr2 (Pimeisl et al., 2013). We find that Tbr2 loss does not alter the dendritic stratification, brain innervation, or cell survival of ipRGCs. However, we do find that Tbr2-deficient RGCs lose melanopsin expression but that ectopic expression of Tbr2 in non-Tbr2^+^ RGCs is not sufficient to induce melanopsin expression. We also find that removal of Tbr2 leads to reduced survival of ipRGCs in an optic nerve injury model. Finally, we show that almost all Tbr2^+^ displaced amacrine cells express Meis2 adding to the molecular definition of this subtype.

## Materials and Methods

### Mice

**FIGURE 1:**
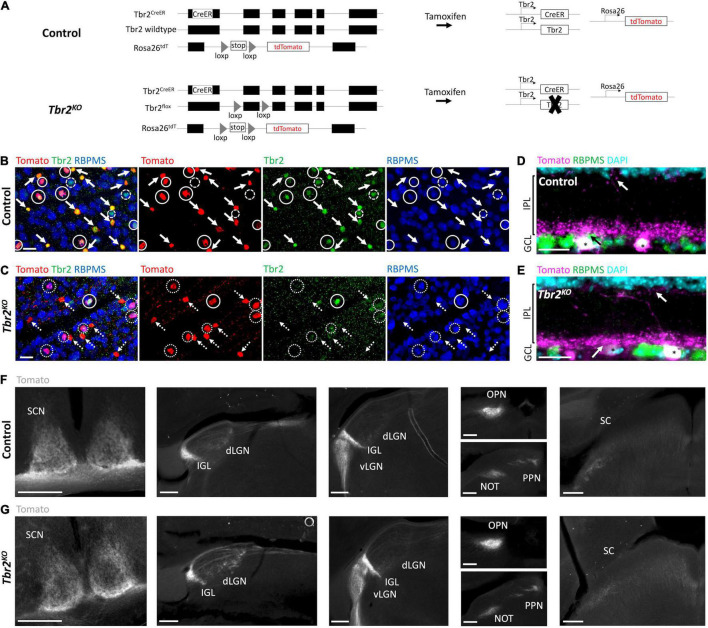
Tbr2*^CreER^* recapitulates endogenous Tbr2 expression and loss of Tbr2 does not alter dendritic stratification or axon projections of Tbr2 expressing neurons. **(A)** Schematic of genetic strategy used to remove Tbr2 from Tbr2^+^ cells in adulthood while simultaneously fluorescently labeling them. Black boxes are exons, thin lines are non-coding regions. **(B,C)** Flatmount view, GCL side up, of a retina derived from a *Tbr2*^CreER/+^*;tdT* (control, **B**) or *Tbr2*^CreER/flox^*;tdT* (*Tbr2^KO^*, **C**) P60 mouse immunostained to reveal expression of Tomato (red), Tbr2 (green), RBPMS (blue) with the first image being a merge of all markers; solid circles represent Tbr2^+^ Tomato-labeled RGCs, solid arrows point to Tbr2^+^ Tomato-labeled amacrine cells (lack RBPMS expression), dashed circles indicate Tbr2^+^ RGCs that do not express Tomato, dotted circles indicate Tomato-labeled RGCs that do not express Tbr2, and dashed arrows point to Tomato-labeled amacrine cells that do not express Tbr2; scale bar = 25 μm. While 98% of wildtype Tomato^+^ RGCs express Tbr2 **(B)**, only 15% express Tbr2 in the mutant **(C)**. **(D,E)** Section of a control **(D)** and *Tbr2^KO^* **(E)** mouse retina immunostained to reveal expression of Tomato (magenta), RBPMS (green), DAPI (cyan); scale bar = 25 μm. There is no difference in the localization of Tomato^+^dendrites between these mice (arrows indicate dendrites of Tomato^+^ RGCs (asterisks) in the innermost and outermost sublaminae of the IPL). **(F,G)** Comparison of the axonal trajectories of Tomato-labeled RGCs in control **(F)** and *Tbr2^KO^* **(G)** mice. Coronal sections reveal that Tomato^+^ RGCs innervate the SCN, dLGN, IGL, vLGN, OPN, PPN, and deep SC in controls **(F)** and maintain this innervation in *Tbr2^KO^* mice **(G)**. Scale bars = 250 μm. GCL, ganglion cell layer; IPL, inner plexiform layer; INL, inner nuclear layer; SCN, suprachiasmatic nucleus; dLGN, dorsal lateral geniculate nucleus; IGL, intergeniculate leaflet; vLGN, ventral lateral geniculate nucleus; OPN, olivary pretectal nucleus; PPN, posterior pretectal nucleus; SC, superior colliculus.

The *Tbr2*^CreER^** (*Eomes^CreER^*) mouse line used in this study was previously described (Pimeisl et al., 2013) (Figure 1). To induce expression of Tbr2*^CreER^*, tamoxifen (Sigma t5648-1G), diluted to 25 mg/mL in corn oil (Sigma c8267), was administered intraperitoneally at a dose of 100 mg/kg of body weight for 3 consecutive days to adult mice. PCR genotyping was performed using the forward primer 5′-GAGGGAGGAAGGGGACATTA-3′ and the reverse primers 5′ CAGGTTCTTGCGAACCTCAT-3′ (to detect Cre) and 5′-AGACTGCCCGGAAACTTCTT-3′ (wildtype allele).

The Tbr2 floxed mouse line (Zhu et al., 2010) was acquired from The Jackson Laboratory (stock no. 017293). PCR genotyping was performed using primers 5′-AGATG GAAATTTGGGAATGAA-3′ and 5′-GGCTACTACGGCCTG AAAC-3′.

The Isl1*^Cre^* mouse line (Srinivas et al., 2001) was acquired from Dr. Eric Ullien (UCSF, Department of Ophthalmology). PCR genotyping was performed using primers 5′-ACCAGAGA CGGAAATCCATCG-3′ and 5′-TGCCACGACCAAGTGACA GCAATG-3′.

The Rosa26-loxp-stop-loxp-tdTomato (Madisen et al., 2010) mouse line was acquired from The Jackson Laboratory (stock no. 007905). PCR genotyping was performed using primers 5′AAGGGAGCTGCAGTGGAGTA-3′ and 5′CCGAAAATCTGTGGGAAGTC-3′ to detect the wildtype allele and primers 5′-CTGTTCCTGTACGGCATGG-3′ and 5′-GGCATTAAAGCAGCGTATCC-3′ to detect the Tomato allele.

C57Bl/6 “wildtype” mice were acquired from The Jackson Laboratory.

Genotyping was performed using genomic DNA extracted from tail clippings using standard techniques.

Both female and male mice were used in this study and no significant differences were observed between them. For each experiment, 3 or more adult mice (P40-P100) were used (number of mice used for each experiment is indicated in the figure legends).

All experimental procedures were performed in accordance with protocols approved by the Institutional Animal Care and Use Committee at the University of California, Santa Cruz.

### Immunohistochemistry and Tissue Processing

Eyes and brains were harvested from mice after intracardial perfusion with phosphate buffered saline (PBS; pH 7.4) followed by perfusion with 4% paraformaldehyde (PFA). For retina wholemount staining, retinas were dissected out of the eye; for retina sections, a hole was made in the cornea prior to fixation. Retinas and eyes were fixed in 4% PFA for 1 h while brains were fixed overnight. Retinas were then transferred to PBS while eyes and brains were transferred to 30% sucrose in PBS. For retina sections, eyes were frozen in Tissue Plus™ O.C.T. compound (Fisher HealthCare) and 20 μm thick sections were obtained via cryostat (Leica cm 3050s) and collected onto SuperFrost Plus slides (Fisher Scientific). For brain tissue, 100 μm thick sections were obtained via a freezing sliding microtome (ThermoFisher microm hm430). For wholemount retinas, retinas were incubated in blocking solution (5% donkey serum, 0.25% TritonX-100 in PBS) for 3 h at room temperature (RT), incubated in primary antibody for 2–3 days at 4°C, washed 3 times (2 h each wash) with 0.1% PBST (PBS with TritonX-100) at RT, incubated in secondary antibody overnight at 4°C, washed 3 times (2 h each wash) with PBS at RT. Immunostained retinas were mounted retinal ganglion cell layer (GCL) side up onto SuperFrost Plus slides where relieving cuts were made. Fluoromount-g tissue mounting medium (SouthernBiotech) was applied prior to coverslipping. For retina sections, slides were incubated in blocking solution for 1 h, incubated in primary antibody overnight at 4°C, washed 3 times (15 min each wash) in PBS at RT, incubated in secondary antibody for 1 h at RT, incubated in DAPI for 10 min, washed 3 times (15 min each wash) in PBS at RT, and lastly covered with fluoromount-g (SouthernBiotech) and coverslipped.

Primary antibodies were diluted in blocking solution at the following concentrations:

Chick anti-GFP (1:1,000; Aves Labs GFP-1020), chick anti-Tbr2 (1:500 flatmount, 1:1,000 sections; Millipore AB15894), rabbit anti-Tbr2 (1:500; Abcam AB183991), rabbit anti-melanopsin (1:1,000; Advanced Targeting Systems AB-N39), goat anti-tdTomato (1:500 flatmount, 1:750 sections; Acris/Sicgen AB8181-200), guinea pig anti-RBPMS (1:250; PhosphoSolutions 1832-RBPMS), mouse anti-meis2 (1:100; DSHB 1A11), rabbit anti-GABA (1:1,000; Sigma A2052).

All secondary antibodies used were diluted 1:1,000 in blocking solution; they are as follows:

AlexaFluor647 donkey anti-guinea pig (Jackson ImmunoResearch AB_2340476 #706-605-148), AlexaFluor594 donkey anti-rabbit (Life Technologies A21207), AlexaFluor555 donkey anti-rabbit (Invitrogen A31572), AlexaFluor555 donkey anti-goat (Invitrogen A21432), AlexaFluor488 donkey anti-mouse (Life Technologies A21202), AlexaFluor488 donkey anti-chick (Jackson ImmunoResearch AB_2340375 #703-545-155), AlexaFluor568 donkey anti-Rabbit (Invitrogen A10042).

### Intravitreal Virus Injection

Mice were anesthetized with isoflurane. This procedure was performed under a dissecting microscope. A hole was created at the corneal-scleral junction with a 26 gauge needle. The vitreous humor was gently massaged out with a cotton swab in order to minimize back-pressure upon injection of virus. A pulled glass pipette preloaded with virus was inserted into the hole and a Picospritzer III (Parker) was used to administer ∼1 μl of virus. One eye in each animal was infected with Tbr2-GFP-AAV2 (Vector Biolabs) while the other was infected with GFP-AAV2 (Vector Biolabs). Retinas were harvested 2 weeks or > 4 weeks after virus injection.

### Optic Nerve Crush

Mice were anesthetized using isoflurane. A ketamine/xylazine cocktail was administered intraperitoneally at a concentration of 100 mg/kg ketamine and 10 mg/kg xylazine. This procedure was performed under a dissecting microscope. Ointment containing atropine sulfate (Bausch and Lomb, NDC 24208-825-55) was applied to both eyes to prevent drying and minimize pain. An incision was made in the sclera using spring scissors (Vannas 3 mm, FST). Subsequently, layers of the eye were gently peeled back using fine forceps (Dumont #55, FST) until the optic nerve was exposed. The optic nerve was crushed ∼2 mm from the posterior pole for 5 s using fine forceps. After the procedure, buprenorphine (0.1 mg/kg of body weight) was administered intraperitoneally and Terramycin ophthalmic ointment (Zoetis) was applied to the experimental eye. Both retinas were harvested 2 weeks after the procedure.

### Data Acquisition

Fluorescent images were obtained with an Olympus BX51 microscope equipped with a Qimaging Retiga EXi Fast 1394 camera or a Zeiss LSM880 confocal microscope. All images presented here were taken with the Olympus microscope.

### Data Analysis and Statistics

Lamination depth of Tomato^+^ AC and RGC dendrites was determined using the IPLaminator plugin (Li et al., 2016) in FIJI. The 20x objective of the Olympus microscope was used to obtain images of retina sections. One or more regions (4000 μm^2^ or greater) from four or more retina sections were analyzed per mouse. Both the “percentile values” (percentile distance across ROI based on measurement of ChAT bands in wildtype mice) and “n Equal boundaries” (ROI divided into 20 equal layers) methods were used to calculate inner plexiform layer (IPL) boundaries.

**FIGURE 2:**
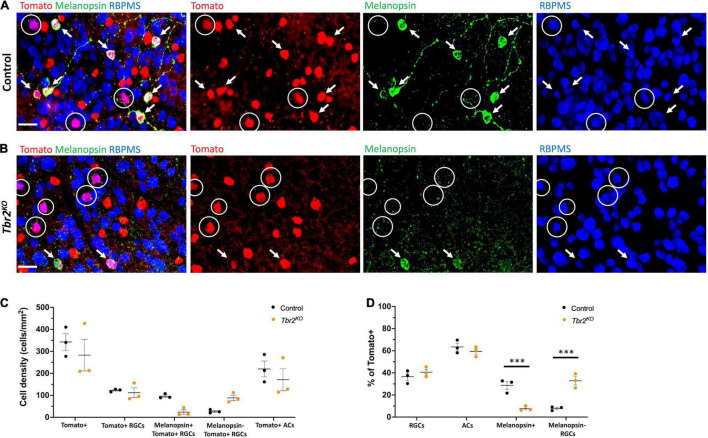
Loss of Tbr2 in adulthood results in loss of melanopsin expression but not cell death of Tomato^+^ RGCs. **(A)** Flatmounted control (*Tbr2^CreER/+^;tdT*) retina (GCL side up) immunostained to reveal expression of Tomato (red), melanopsin (green), and RBPMS (blue); circled cells are Tomato^+^ RGCs that do not express melanopsin, arrows point to melanopsin^+^Tomato^+^ RGCs, scale bar = 25 μm. **(B)** Flatmounted *Tbr2*^KO^** (*Tbr2*^CreER/flox^*;tdT*) retina immunostained to reveal expression of Tomato (red), melanopsin (green), and RBPMS (blue); circled cells are Tomato^+^ RGCs that do not express melanopsin, arrows point to melanopsin^+^Tomato^+^ RGCs. Tomato^+^ cells lose melanopsin expression. **(C)** Quantification of cell densities (cells/mm^2^) of different cell populations in control and *Tbr2*^KO^** mice (*n* = 3 mice in each group; each dot represents the sum of four fields of view (FOVs) for 1 retina; mean ± SEM displayed; two-way ANOVA with Sidak’s multiple comparisons test; no significant differences). **(D)** Quantification of the percentages of Tomato^+^ cells that belong to cell populations of interest in control and *Tbr2*^KO^** mice (*n* = 3 mice in each group; each dot represents the percentage for 4 summed FOVs of 1 retina; mean ± SEM displayed; two-way ANOVA with Sidak’s multiple comparisons test; *** *P* < 0.001).

**FIGURE 3:**
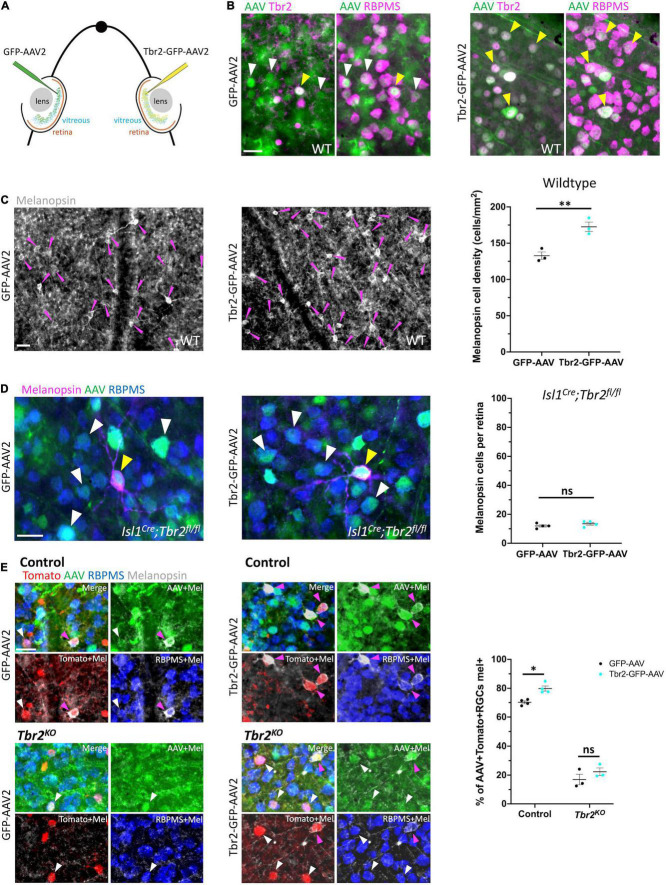
Ectopic expression of Tbr2 does not induce melanopsin expression in non-Tbr2 RGCs. **(A)** Experimental overview of intravitreal AAV2 injection. One eye of an adult mouse (∼P40) was injected with a control virus, GFP-AAV2, while the contralateral eye was injected with a Tbr2-expressing virus, Tbr2-GFP-AAV2. **(B)** Flatmounted retinas of eyes injected with GFP-AAV2 (left) or Tbr2-GFP-AAV2 (right) are immunostained with the antibody indicated: Tbr2 (magenta), GFP (green), and RBPMS (magenta); the left images for each virus condition are showing overlap of GFP and Tbr2 expression while the right images are showing overlap of GFP and RBPMS expression; white arrowheads indicate example virus-infected RGCs that do not express Tbr2 and yellow arrowheads indicate example virus-infected RGCs that express Tbr2; scale bar = 25 μm. In the Tbr2-GFP-AAV2-infected retina (right), all virus-infected RGCs express Tbr2. **(C)** Flatmounted retinas of a wildtype mouse infected with GFP-AAV2 (left) and Tbr2-GFP-AAV2 (middle) and immunostained to reveal expression of melanopsin (grayscale); magenta arrowheads point to melanopsin^+^ cells, scale bar = 25 μm. Right panel is showing the quantification of melanopsin^+^ cells in these retinas (*n* = 3 mice for each group; each dot represents the sum of 4 FOVs per retina; mean ± SEM displayed; Student’s *t*-test; **P < 0.01). There is a significant increase in melanopsin^+^ cells in the Tbr2-GFP-AAV2-infected retina. **(D)** Flatmounted retinas of an *Isl1*^Cre^*;Tbr2*^flox/flox^** mouse infected with GFP-AAV2 (left) and Tbr2-GFP-AAV2 (middle) immunostained to reveal expression of melanopsin (magenta), GFP (green), RBPMS (blue); white arrowheads point to example melanopsin-negative RGCs infected with virus, yellow arrowheads point to rare melanopsin^+^ cells, scale bar = 25 μm. Right panel is showing the quantification of melanopsin^+^ cells (*n* = 4 mice in each group; each dot represents the total number of melanopsin^+^ cells in 1 retina; mean ± SEM displayed; Student’s *t*-test; ns = *P* > 0.05). There is no change in melanopsin expression in Tbr2-GFP-AAV2-infected retinas in *Isl1*^Cre^*;Tbr2*^flox/flox^** mice. **(E)** Flatmounted retinas of control (*Tbr2^CreER/+^;tdT*, top) and *Tbr2*^KO^** (*Tbr2*^CreER/flox^*;tdT*, bottom) mice immunostained to reveal tomato (red), GFP (green), RBPMS (blue), and melanopsin (gray) expression in GFP-AAV2-infected (left) and Tbr2-GFP-AAV2-infected (middle) retinas. The first image of each is a merge of all color channels, others are showing melanopsin in combination with the other markers indicated. Magenta arrowheads point to virus-infected Tomato^+^ RGCs that express melanopsin and white arrowheads point to virus-infected Tomato^+^ RGCs that do not express melanopsin. Scale bar = 25 μm. Right, quantification of the percentage of virus-infected Tomato^+^ RGCs that express melanopsin in control and *Tbr2*^KO^** mice infected with GFP-AAV2 and Tbr2-GFP-AAV2 (*n* = 4 mice for each group, each dot represents the percentage for the sum of 7 or more FOVs in one retina; mean ± SEM displayed; two-way ANOVA with Sidak’s multiple comparisons, *P < 0.05, ns = *P* > 0.05). There is increased melanopsin expression in Tbr2-GFP-AAV2-infected Tomato^+^ RGCs.

**FIGURE 4:**
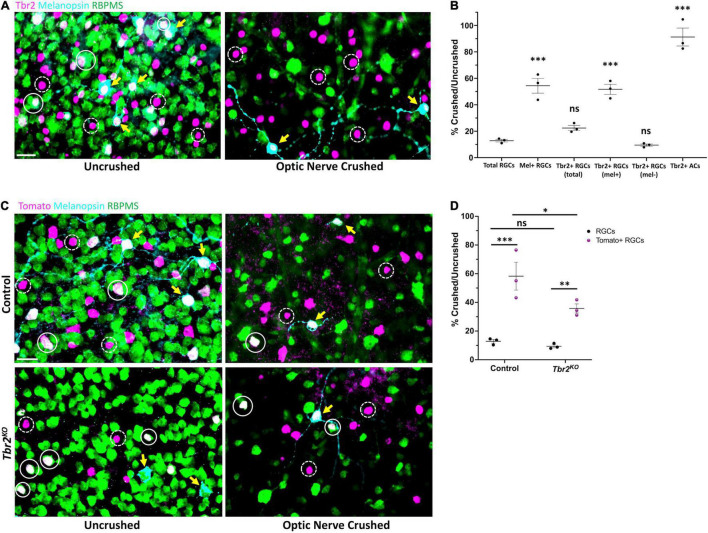
Tbr2-expressing RGCs preferentially survive optic nerve injury and Tbr2 influences their survival. **(A)** Flatmounted retinas of a wildtype mouse 2 weeks after optic nerve crush in one eye (right, “optic nerve crushed”). The optic nerve of the contralateral control eye was not crushed (left, “uncrushed”). Both retinas were stained to reveal expression of Tbr2 (magenta), melanopsin (cyan), and RBPMS (green); solid white circles represent Tbr2^+^ RGCs that do not express melanopsin, yellow arrows point to melanopsin^+^Tbr2^+^ RGCs, dashed white circles indicate Tbr2^+^ amacrine cells, scale bar = 25 μm. **(B)** Quantification of percent survival of cell populations of interest in ONC eyes normalized to uncrushed control eyes (*n* = 3 mice, each dot represents the percentage for one mouse-4 FOVs/retina; mean ± SEM displayed; one-way ANOVA with Dunnett’s multiple comparisons, comparing each group to RGCs; ***P < 0.001, ns = *P* > 0.05). A significantly greater percentage of melanopsin^+^Tbr2^+^ RGCs survive ONC compared to other RGCs. **(C)** Left, flatmounted retinas of uncrushed control (*Tbr2^CreER/+^;tdT*, top) and *Tbr2*^KO^** (*Tbr2*^CreER/flox^**, bottom) eyes. Right, flatmounted retinas of optic nerve crushed control (top) and *Tbr2*^KO^** (bottom) eyes immunostained to reveal expression of Tomato (magenta), melanopsin (cyan), and RBPMS (green). Scale bar = 25 μm. **(D)** Quantification of percent survival of Tomato^+^ RGCs (purple, pattern) and all other RGCs (black, solid) in ONC eyes normalized to uncrushed control eyes in control and *Tbr2*^KO^** mice (*n* = 3 mice for each group, each dot represents the percentage for one mouse-4 FOVs/retina; mean ± SEM displayed; two-way ANOVA with Sidak’s multiple comparisons, ***P < 0.001, **P < 0.01, *P < 0.05, ns *P* > 0.05). Tomato^+^ RGCs have increased survival relative to other RGCs in both control and *Tbr2*^KO^** mice but show decreased survival in *Tbr2*^KO^** mice.

**FIGURE 5:**
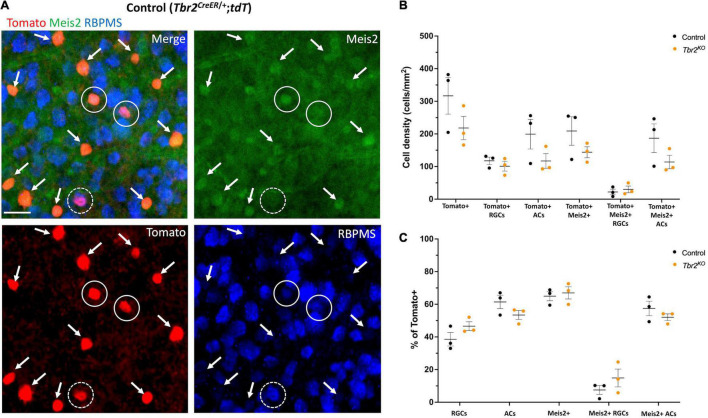
Meis2 is expressed in the majority of Tbr2-expressing displaced amacrine cells and in a subset of Tbr2-expressing RGCs. **(A)** Flatmounted retina of a control (*Tbr2^CreER/+^;tdT)* mouse immunostained to reveal expression of tomato (red), Meis2 (green), and RBPMS (blue). First image is a merge of all color channels, others are showing each channel independently; arrows point to Tomato^+^ ACs expressing Meis2; closed white circles represent Tomato^+^ RGCs expressing Meis2; dashed white circles indicate Tomato^+^ RGCs that do not express Meis2. All Tomato^+^ ACs in this image express Meis2. **(B)** Quantification of the density (cells/mm^2^) of cell populations of interest in control and *Tbr2*^KO^** mice (*n* = 3 mice in each group, each dot represents the sum of 4 FOVs per retina, mean ± SEM displayed; two-way ANOVA with Sidak’s multiple comparisons test; no significant differences). There is a trend toward a decrease in the density of labeled amacrine cells in *Tbr2*^KO^** mice. **(C)** Quantification of the percentage of Tomato^+^ cells that belong to cell populations of interest in control and *Tbr2*^KO^** mice (*n* = 3 mice in each group; each dot represents the percentage for 4 summed FOVs of 1 retina; mean ± SEM displayed; two-way ANOVA with Sidak’s multiple comparisons test; no significant differences).

For cell density analyses, cells were manually counted in FIJI using the multi-point tool. For Figures 2, 3C, 4, four fields of view (446.15 μm × 333.33 μm) were imaged with the 20x objective of the Olympus microscope. One image was taken per retina quadrant at approximately the same distance from the center of the retina (between ∼1.5 and 3.5 mm from center). For Figure 3D, the entire retina was analyzed as there are very few melanopsin cells in the *Isl1*^Cre^*;Tbr2*^flox/flox^** mice. For Figure 3E, seven or more fields of view (446.15 μm × 333.33 μm) were imaged. For Figures 5B,C, four fields of view (425.1 × 425.1 μm) were imaged with the 20x objective of the Zeiss confocal microscope.

Statistical analyses and graph generation were performed using GraphPad’s Prism 9 software. Statistical tests used and number of animals used are indicated in the figure legends. In experiments where multiple cell populations were compared in more than one genotype (*Tbr2^CreER/+^;tdT* vs. *Tbr2*^CreER/flox^*;tdT*), two-way ANOVA was performed with Sidak’s multiple comparisons *post-hoc* test. In experiments where multiple cell populations were compared in a single genotype, one-way ANOVA was performed. Student’s *t*-test was performed for comparisons between two groups.

## Results

### Tbr2 Is Required for Maintaining Melanopsin Expression in Intrinsically Photosensitive Retinal Ganglion Cells

To test the hypothesis that Tbr2 is required in adulthood for ipRGC survival, we removed *Tbr2* from adult Tbr2-expressing RGCs using *Tbr2*^CreER/flox^*;tdT* mice. These mice have a tamoxifen-inducible Cre recombinase inserted into exon 1 of the *Tbr2* locus (*Tbr2*^CreER^**; Pimeisl et al., 2013), a *Tbr2* floxed allele (*Tbr2^flox^*; Zhu et al., 2010), and a Rosa26-tdTomato reporter (*tdT*; Madisen et al., 2010). Tamoxifen administration to *Tbr2*^CreER/flox^*;tdT* mice results in coincident fluorescent labeling and Tbr2 removal in CreER-expressing cells (Figure 1A). We administered tamoxifen to adult *Tbr2^CreER/+^;tdT* (hereafter “control”)*and Tbr2*^CreER/flox^*;tdT* (hereafter “*Tbr2*^KO^**”) animals and asked if loss of *Tbr2* results in Tbr2-expressing RGC death, as happens when *Tbr2* is deleted during development, and if not, whether Tbr2 is required for specifying their axonal projections or dendrite stratification patterns. First we verified that Tbr2*^CreER^* labels endogenous Tbr2-expressing RGCs by immunostaining retinas derived from control mice with antibodies directed against Tbr2 and RBPMS (a pan-RGC marker; Rodriguez et al., 2014) and determining the percent overlap of these markers with Tomato fluorescence (Figure 1B). We find that all Tomato-labeled cells are also labeled with an anti-Tbr2 antibody, illustrating that Tbr2*^CreER^* recapitulates endogenous Tbr2 expression (Figure 1B). We also find that 63% ± 3 (393 cells, *n* = 3 mice) of Tomato-expressing cells do not express RBPMS, corroborating the recent finding that these cells are displaced amacrine cells (ACs; Chen et al., 2021, see below). The majority of Tomato-expressing cells in the ganglion cell layer (GCL) have dendrites that laminate in the innermost ON sublamina of the inner plexiform layer (IPL) and sparsely in the outermost OFF sublamina (Figure 1D and Supplementary Figure 1), consistent with displaced AC and ipRGC lamination patterns (Provencio et al., 2002b; Viney et al., 2007; Schmidt and Kofuji, 2009; Ecker et al., 2010; Quattrochi et al., 2018; Chen et al., 2021).

Because the Tomato labels the axons of RGCs, we can also determine where Tbr2-expressing axons project in the brain. Tomato-expressing RGCs project to all brain regions known to be innervated by ipRGCs including the SCN, external vLGN, IGL, OPN, PPN, as well as sparsely to the dLGN and deep SC (Figure 1F).

We then aimed to determine if loss of *Tbr2* in adulthood affects the survival or health of Tbr2^+^ RGCs by administering tamoxifen to *Tbr2*^KO^** mice and analyzing (as above) the retina and brain targets 33–36 days later. We find that Cre activation results in the loss of expression of Tbr2 in most Tomato-expressing RGCs (85% ± 3, 162/189 Tomato^+^ RGCs, *n* = 3 mice; Figure 1C). However, removing Tbr2 does not affect the overall number of Tomato-expressing RGCs compared to controls (112 ± 22 cells/mm^2^ in *Tbr2*^KO^** mice, 200 Tomato^+^ RGCs counted vs. 122.7 ± 3 cells/mm^2^ in control mice, 219 Tomato^+^ RGCs counted, *n* = 3 mice for each genotype, *P* = 0.9998; Figures 1B,C, 2C). Analysis of retina sections (*n* = 3 mice) shows that Tbr2-deficient RGC and AC dendrites continue to laminate within the innermost ON and sparsely in the outermost OFF sublaminae of the IPL (Figure 1E and Supplementary Figure 1). We also find that Tomato-labeled RGC axons in mutant mice maintain projections to their brain targets and innervate them to a similar extent as in control mice (Figure 1G). Taken together these results demonstrate that *Tbr2* is not required for the maintenance of dendrite localization, axon projections, or RGC survival.

Melanopsin expression is a key feature of ipRGCs therefore we next asked whether Tbr2 is required for this aspect of ipRGC identity. We stained control and *Tbr2*^KO^** retinas with an anti-melanopsin antibody (Figures 2A,B) and found that there is a 75% reduction in melanopsin-expressing Tomato-labeled cells in *Tbr2*^KO^** retinas relative to control retinas (42/200 Tomato^+^ RGCs vs. 171/219 Tomato^+^ RGCs; Figure 2C) derived from littermates. However, as previously mentioned, there is no difference in the number of Tomato-expressing RGCs in the mutant retinas. Because there is some variation in the proportion of Cre-activated cells in each mouse, we also looked at the percentage of Tomato-labeled cells belonging to cell populations of interest (Figure 2D). We find a significant reduction in the percentage of Tomato-labeled cells that also express melanopsin (8% ± 1 vs. 29% ± 3, *P* < 0.001, 505 Tomato^+^ cells and 612 Tomato^+^ cells scored per genotype, respectively) and a significant increase in the percentage of Tomato-labeled cells that are non-melanopsin-expressing RGCs (33% ± 4 vs. 8% ± 1, *P* < 0.001) in *Tbr2*^KO^** mice relative to control mice (Figure 4D). Together, these data illustrate that while melanopsin expression is lost in Tbr2-mutant RGCs, their survival is unaffected. This result conflicts with two recent studies that reported a decrease in survival of melanopsin and/or Tbr2-expressing RGCs after conditional removal of *Tbr2* using different Cre systems. One study showed a ∼50% reduction in the number of ipRGCs ∼40 days after conditional Tbr2 removal (Bray et al., 2019) and the other found a near complete loss of Tbr2-expressing cells 38 days after conditional Tbr2 removal (Chen et al., 2021). We worried that one difference in these studies compared to ours is the time after Cre activation (∼40 vs. ∼30 days, respectively). To address this discrepancy, we performed a separate experiment in which we waited 45–50 days before analysis. We found that the number of Tomato-expressing RGCs in *Tbr2*^KO^** mice remained similar to controls (101 ± 15 cells/mm^2^, 219 Tomato^+^ RGCs counted vs. 117 ± 11 cells/mm^2^, 255 Tomato^+^ RGCs counted, respectively, *n* = 3 for each genotype, *P* = 0.9995; Figure 5B). However, there appears to be a trending, but not statistically significant, decrease of Tomato-expressing amacrine cells (117 ± 22 cells/mm^2^, 254 Tomato^+^ ACs counted in *Tbr2*^KO^** mice and 199 ± 45 cells/mm^2^, 432 Tomato^+^ ACs counted in control mice, *n* = 3 mice for each genotype, *P* = 0.39; Figure 5B).

### Tbr2 Is Not Sufficient for Melanopsin Expression

To test whether ectopic expression of Tbr2 can induce expression of melanopsin, we intravitreally injected Tbr2-GFP-AAV2 or GFP-AAV2 into the eyes of wildtype adult mice and examined expression of markers 4 weeks later (Figure 3A). First, to determine if infection of RGCs with Tbr2-GFP-AAV2 results in production of Tbr2 protein, we performed intravitreal virus injection with AAV during adulthood (∼P40), waited 2 weeks, then dissected retinas and examined Tbr2 expression. We find that all Tbr2-GFP-AAV2-infected cells also express Tbr2, while 17% of cells infected with GFP-AAV2 express Tbr2 (Figure 3B). Interestingly, this is higher than the percent of Tbr2-expressing RGCs in wildtype retina (11.3% ± 0.3, 596 Tbr2^+^ RGCs/5,279 total RGCs, *n* = 3 mice, uncrushed eyes from Figure 4B; consistent with Chen et al., 2021), suggesting that Tbr2-expressing RGCs are preferentially infected with the virus. We also find that there is increased density of melanopsin-expressing RGCs in Tbr2-GFP-AAV2-infected retinas relative to GFP-AAV2-infected retinas 4 weeks after AAV injection (173 ± 6 cells/mm^2^, 308 melanopsin cells counted vs. 133 ± 5 cells/mm^2^, 237 melanopsin cells counted, *n* = 3 mice, *P* = 0.00855; Figure 3C). However, we find that many Tbr2-GFP-AAV2-infected RGCs do not express melanopsin (89%, 662 cells, Supplementary Figure 2) leading to the hypothesis that this increase in ipRGC density is the result of over-expressing Tbr2 in cells that already express Tbr2, but do not express melanopsin at levels sufficient to be detected by melanopsin antibody. To determine whether expression of Tbr2 can induce melanopsin expression in non-Tbr2-expressing RGCs, we injected Tbr2-GFP-AAV2 and GFP-AAV2 into the eyes of *Isl1^Cre/+^;Tbr2^flox/flox^* mice. Isl1-Cre removes *Tbr2* during development and leads to cell death, thus these mice lack > 99% of ipRGCs as assayed by melanopsin expression (Supplementary Figure 3) and loss of axon projections to non-image-forming brain targets. In these mice, we do not observe an increase in melanopsin expression in the Tbr2-GFP-AAV2-infected retinas (Figure 3D), indicating that Tbr2 is not sufficient for melanopsin expression in non-Tbr2^+^ RGCs (13.5 ± 0.96 cells per Tbr2-GFP-AAV2-infected retina vs. 12 ± 0.82 cells per GFP-AAV2-infected retina, *n* = 4 mice, *P* = 0.2782). To determine whether melanopsin expression is only induced in endogenous Tbr2 cells, we performed the same experiment in control (*Tbr2^CreER/+^;tdT)* and *Tbr2*^KO^** (*Tbr2*^CreER/flox^*;tdT)* mice after tamoxifen induction to label endogenous Tbr2 cells with tdTomato. We find a significant increase in the percentage of Tbr2-expressing RGCs that also express melanopsin in the Tbr2-GFP-AAV2-infected retinas relative to retinas infected with control virus (80% ± 2 vs. 70% ± 1, *n* = 4 mice, *P* = 0.0156, 259 Tbr2-GFP-AAV2-infected Tomato^+^ RGCs scored and 301 GFP-AAV2-infected Tomato^+^ RGCs scored; Figure 3E), but there is not a significant increase when Tbr2 is absent (22% ± 3 vs. 17% ± 4, *n* = 3 mice, *P* = 0.2651, 231 Tbr2-GFP-AAV2-infected Tomato^+^ RGCs scored and 172 GFP-AAV2-infected Tomato^+^ RGCs scored; Figure 3E). These data show that Tbr2 can increase melanopsin expression in a subset of Tbr2-expressing RGCs but cannot if Tbr2 is deleted.

### Tbr2 Expression Influences Intrinsically Photosensitive Retinal Ganglion Cell Survival After Injury

ipRGCs preferentially survive after optic nerve injury relative to RGCs that are not intrinsically photosensitive (Robinson and Madison, 2004; Li et al., 2008; Pérez De Sevilla Müller et al., 2014; Duan et al., 2015), yet the reason for this is poorly understood. Because Tbr2 is enriched in surviving RGCs (Tran et al., 2019), we hypothesized that Tbr2 could be required for RGC survival after optic nerve crush (ONC). First we wanted to determine whether all Tbr2-expressing RGCs survive ONC or if only the melanopsin-expressing subset of Tbr2-expressing RGCs is spared. To test this, we performed ONC on one eye of an adult (∼P60) mouse, waited 2 weeks, then examined both retinas for expression of RGC markers. In the uncrushed eye, 11.3% ± 0.3 (*n* = 3 mice, 596 cells) of RBPMS-labeled cells express Tbr2 and 30.9% ± 2.1 (186 cells) of Tbr2-expressing RGCs express melanopsin (the melanopsin-negative Tbr2-expressing RGCs are likely M4-M6 ipRGCs that express low levels of melanopsin; Ecker et al., 2010; Quattrochi et al., 2018). In the ONC retinas, we find that 13% ± 1 (670 cells; normalized to control “uncrushed” eye) of RGCs survive (Figures 4A,B). We also find that 22% ± 2 (134 cells) of total Tbr2-expressing RGCs survive. The majority of these surviving cells express melanopsin (71% ± 1; 95 cells), suggesting that the melanopsin-expressing Tbr2-expressing RGCs preferentially survive optic nerve injury (Figure 4B). When separating Tbr2-expressing RGCs into those that express melanopsin and those that do not, we find that 52% ± 4 (95 cells, *P* < 0.001, significantly greater than survival of all RGCs: 13% ± 1) and 10% ± 1 (39 cells) survive, respectively (Figure 4B). There is no change in the survival of Tbr2-expressing amacrine cells (Figure 4B) indicating that they are unaffected by ONC, consistent with previous reports regarding amacrine cell survival after optic nerve injury (Nadal-Nicolás et al., 2015).

To test if Tbr2 expression is required for the preferential survival of Tbr2-expressing RGCs, we performed ONC in control (*Tbr2^CreER/+^;tdT*) and *Tbr2*^KO^** (*Tbr2*^CreER/flox^*;tdT*) mice (Figures 4C,D). We find that in control mice, 58% ± 10 (100 cells crushed, 183 cells uncrushed) of Tomato-expressing RGCs survive nerve crush while only 13% ± 1 (711 cells crushed, 6,058 cells uncrushed) of non-Tomato-expressing RGCs survive (*P* < 0.001; Figures 4C,D). However, in *Tbr2*^KO^** mice only 35% ± 3 (70 cells crushed, 199 cells uncrushed) of Tomato-expressing RGCs survive (*P* = 0.0153, compared to survival in control mice) while 9% ± 1 (508 cells crushed, 5,544 cells uncrushed) of non-Tomato-expressing RGCs survive (*P* < 0.01; Figures 4C,D) suggesting that Tbr2 influences, but is not essential for, ipRGC survival after injury.

### Tbr2^+^ Cells in the GCL Include Displaced Amacrine Cells That Express the GABAergic Amacrine Cell Marker, Meis2

As noted earlier, we find that 63% of Tbr2-expressing cells in the GCL of the adult retina do not express the RGC marker RBPMS, suggesting that these are displaced amacrine cells. It has been previously reported that some Tbr2-expressing cells also express the pan-amacrine cell marker syntaxin-1 (Mao et al., 2008), and more recently that over half of the Tbr2-expressing cells in the GCL are displaced amacrine cells labeled in the *slc32aiCre;Ai9* mouse strain (Chen et al., 2021). Most displaced amacrine cells are GABAergic (Pérez De Sevilla Müller et al., 2007). However, we previously looked at the degree of GABA and Tbr2 co-expression in P8 retinas and found that only 3% of Tbr2 cells in the GCL express GABA (Sweeney et al., 2014). Recently, Meis2, a GABAergic amacrine cell marker (Bumsted-O’Brien et al., 2007), was found to be expressed in Tbr2^+^ cells in the inner nuclear layer (INL) of mouse retina (Yan et al., 2020), therefore we revisited this question. To determine whether Meis2 is also expressed in displaced Tbr2-expressing amacrine cells, we treated retinas from adult tamoxifen-induced *Tbr2^CreER/+^;tdT* mice (45–50 days after tamoxifen administration) with an anti-Meis2 antibody and found that Meis2 is expressed in 93% ± 2 (405 cells) of Tbr2-expressing cells that do not express RBPMS in the GCL (Figures 5A,B). Meis2 is also expressed in a subset of Tbr2-expressing RGCs (18% ± 11, 48 cells). We then asked whether Tbr2 is required for Meis2 expression or maintenance of Meis2-expressing cells. In adult *Tbr2*^KO^** mice, we found that Meis2 expression was unchanged; the percentage of Tbr2-expressing cells that also express Meis2 is not significantly different in *Tbr2*^KO^** mice relative to control mice (67% ± 4 vs. 65% ± 3, *P* = 0.9978, 473 Tomato^+^ cells scored and 687 Tomato^+^ cells scored, respectively; Figure 5C). Additionally, there is no difference in the percentage of Tomato-labeled cells that are Meis2-expressing ACs (52% ± 2 in *Tbr2*^KO^** mice and 57% ± 4 in control mice, *P* = 0.8399) or Meis2-expressing RGCs (15% ± 5 in Tbr2*^KO^* mice and 8% ± 2 in control mice, *P* = 0.6008). However, there does appear to be a trend toward a reduction in the number of Tomato^+^ amacrine cells in the mutant mice (Figures 5B,C). We immunostained wildtype retinas with anti-Meis2 antibody and the anti-GABA antibody that was used in our previous study (Sweeney et al., 2014) to determine whether Meis2 cells express GABA (Supplementary Figure 4). We find that hardly any cells in the GCL are labeled by the anti-GABA antibody (Supplementary Figure 4C), contrary to previous findings using a different anti-GABA antibody (Pérez De Sevilla Müller et al., 2007). Additionally, many Meis2-expressing cells in the INL are not labeled by the anti-GABA antibody while it has been shown that the majority of Meis2-expressing cells in the INL express GAD65/67 (Bumsted-O’Brien et al., 2007), the GABA synthesizing enzymes. We suspect that this particular anti-GABA antibody does not reliably identify GABAergic cells with our immunostaining technique and thus our previous conclusion that Tbr2 cells do not express GABA was misconceived.

## Discussion

Here we use a tamoxifen-inducible Cre line to examine the role of Tbr2 in the adult mouse retina. Previous work from our lab and others have shown that *Tbr2* is required for the development of ipRGCs (Mao et al., 2014; Sweeney et al., 2014), but its role in the adult retina has only recently been explored (Bray et al., 2019; Chen et al., 2021). In our present study, we show that Tbr2 is required for the maintenance of melanopsin expression in ipRGCs but is dispensable for their survival. Additionally, we find that Tbr2 induces melanopsin expression in endogenous Tbr2 RGCs but is unable to do so in conventional RGCs nor can it restore melanopsin expression in Tbr2-mutant RGCs. Furthermore, we show that Tbr2-expressing ipRGCs survive after optic nerve injury and that this resilience is diminished in Tbr2-mutant RGCs. Lastly, we find a marker for Tbr2-expressing displaced amacrine cells that also labels a subset of Tbr2-expressing RGCs.

### Tbr2 Is Not Required for the Survival of Mature Intrinsically Photosensitive Retinal Ganglion Cells

Our results showing that conditional deletion of Tbr2 in the adult does not affect RGC survival is contrary to what others have reported using different methods. Bray et al. (2019) concluded that Tbr2 is required for the maintenance of ipRGC viability. They used Opn4*^CreER^* to remove a conditional Tbr2 allele while labeling the mutant cells with a tdTomato fluorescent reporter (Bray et al., 2019). They reported that ∼40 days after tamoxifen administration there was a ∼50% reduction in the number tdTomato-expressing cells compared to controls, while we find no significant differences (Figures 2, 5). One possible explanation for the difference between this result and ours comes from the different methods to remove adult Tbr2 expression. Because Opn4 expression is dependent upon Tbr2 expression (Figure 2), the removal of Tbr2 in adult RGCs should result in a loss of melanopsin expression and thus Opn4*^CreER^* expression. This would lead to a decrease of tdTomato-labeled RGCs because once Tbr2 expression is removed no new cells can become activated. A second recent study by Chen et al. (2021) removed Tbr2 via intravitreal injection of AAV-Cre in *Tbr2^TauGFP–IRESCreER2/fx^* mice. They found that 12 days after injection, Tbr2 expression was lost as assayed by Tbr2 antibody staining, but the cells survived (labeled by GFP via *Tbr2^TauGFP–IRESCreER2^*; Chen et al., 2021). However, 38 days after injection, there were few GFP-expressing RGCs in Tbr2-deleted regions. In this model, GFP expression relies on the expression of Tbr2. One hypothesis for their observations is that Tbr2 regulates its own gene expression and once Tbr2 is removed it can no longer activate GFP expression. Consistent with this, Chip-seq experiments show that Tbr2 binds its own locus in E14.5 mouse cortex and has been identified as a direct activator of *Tbr2* (Sessa et al., 2017; Elsen et al., 2018; Hevner, 2019).

### Tbr2 Regulates Adult Melanopsin Expression

While removal of Tbr2 in adult mice leads to the loss of melanopsin expression (Figure 2), ectopic expression of Tbr2 only induces melanopsin expression in Tbr2-expressing RGCs. When Tbr2 was ectopically expressed in the retinas of wildtype mice, we observed a modest increase (30% ± 8) in the number of melanopsin-expressing cells (Figure 3C). However, when we did the same experiment in ipRGC-deficient mice (*Isl1*^Cre^*;Tbr2*^flox/flox^**), we did not detect an increase of melanopsin expressing RGCs (Figure 3D). This result could be explained if only the endogenously Tbr2-expressing RGCs can change their melanopsin expression upon Tbr2 addition. Consistent with this, ectopic expression of Tbr2 in *Tbr2^CreER/+^;tdT* mice but not in *Tbr2*^CreER/fl^*;tdT* mice resulted in increased melanopsin expression in Tomato-labeled RGCs (Figure 3E). Lower levels of expression of Tbr2 could be one reason why M4-M6 ipRGC types express less melanopsin than M1-M3 ipRGC types (Ecker et al., 2010; Quattrochi et al., 2018).

### Tbr2 Mutant Retinal Ganglion Cells Have Reduced Survival After Injury

Although ipRGCs preferentially survive after optic nerve crush, the reason for their survival is unknown. In this study we find that Tbr2 mutant RGCs (which lack melanopsin expression) do not survive as well as their wildtype counterparts after nerve crush. This result is consistent with what was reported by Bray et al. (2019) in which they reported a 30% reduction in Tomato-expressing cell survival after optic nerve crush in Tbr2 mutants (*Opn4*^CreER/+^*;Tbr2*^flox/flox^*;tdT*) relative to wildtype (*Opn4*^CreER/+^*;Tbr2^+/+^;tdT)*. They further showed that lack of melanopsin expression alone does not account for the survival difference leading to the hypothesis that Tbr2 regulates non-melanopsin genes that are involved in ipRGC survival after injury, such as PACAP (Seki et al., 2008; Ye et al., 2019), but this remains to be determined.

### Meis2 Labels the Majority of Tbr2^+^ Displaced ACs and a Subset of Tbr2^+^ RGCs

It has been previously shown that all Tbr2-expressing cells in the inner nuclear layer of the retina also express Meis2 (Yan et al., 2020) but the expression of Meis2 in cells within the GCL has not yet been explored. Here we show that Meis2 is expressed in the majority of Tbr2-expressing displaced amacrine cells and in a subset of Tbr2-expressing RGCs. Using the Broad Institute’s Single Cell Portal to explore the single cell sequencing dataset acquired in the aforementioned study (Yan et al., 2020), we find that Tbr2 is expressed in 6 out of 63 uniquely identified clusters of amacrine cells out of 63 total clusters identified (clusters # 44, 48, 54, 57, 59, 63). Of these 6 clusters, all express Gad1 and Gad2 (GABA synthesis enzymes) and all but 1 cluster (#57) express Meis2. This non-Meis2-expressing cluster accounts for the 7% of displaced Tbr2-expressing ACs that were not labeled by the Meis2 antibody in this study. Using the same tool to explore RGC RNA-sequencing datasets (Tran et al., 2019), we find that Tbr2 is expressed in 8 clusters (clusters # 7, 8, 22, 29, 31, 33, 40, 43) which include all of those that express melanopsin (7-low, 8-low, 22, 31, 33, 40, 43). Of the clusters expressing Tbr2, #s 29 and 40 express Meis2. Cluster 29 corresponds to the only cluster of Tbr2-expressing RGCs that does not express melanopsin and is a “novel” cluster, indicating that no known RGC subtypes correspond to this cluster. It would be interesting to determine whether this group is the newly identified Tbr2-expressing Pou4f1/Brn3a OFF RGC subtype (Chen et al., 2021). Cluster 40 corresponds to M1 cells which also express Gad2 according to this dataset. These Tbr2-expressing Meis2-expressing M1 cells are likely the recently identified GABAergic subset of ipRGCs (Sonoda et al., 2020).

## Conclusion

In conclusion, these findings demonstrate several important roles of the transcription factor Tbr2 in the mature retina: its requirement for melanopsin expression in ipRGCs, and therefore for the maintenance of ipRGC identity; its ability to activate melanopsin expression in endogenous Tbr2 cells; and its involvement in ipRGC survival after optic nerve injury.

## Data Availability Statement

The raw data supporting the conclusions of this article will be made available by the authors, without undue reservation.

## Ethics Statement

The animal study was reviewed and approved by the UCSC IACUC.

## Author Contributions

DF, AR, and SA conceived of and designed experiments. AR performed initial experiments not included in the manuscript. SA performed all experiments included in the manuscript, analyzed and interpreted the data, and wrote the manuscript with support from DF. SJA provided the *Tbr2*^CreER^** mice. All authors discussed and commented on the results and approved of the submitted version of the manuscript.

## Conflict of Interest

The authors declare that the research was conducted in the absence of any commercial or financial relationships that could be construed as a potential conflict of interest.

## Publisher’s Note

All claims expressed in this article are solely those of the authors and do not necessarily represent those of their affiliated organizations, or those of the publisher, the editors and the reviewers. Any product that may be evaluated in this article, or claim that may be made by its manufacturer, is not guaranteed or endorsed by the publisher.

## References

[B1] Agorastos A. Skevas C. Matthaei M. Otte C. Klemm M. Richard G. (2013). Depression, Anxiety, and Disturbed Sleep in Glaucoma. *J. Neuropsychiatry Clin. Neurosci.* 25 205–213. 24026713 10.1176/appi.neuropsych.12020030

[B2] Baden T. Berens P. Franke K. Román Rosón M. Bethge M. Euler T. (2016). The functional diversity of retinal ganglion cells in the mouse. *Nature* 529 345–350. 10.1038/nature16468 26735013 PMC4724341

[B3] Bae J. A. Mu S. Kim J. S. Turner N. L. Tartavull I. Kemnitz N. (2018). Digital Museum of Retinal Ganglion Cells with Dense Anatomy and Physiology. *Cell* 173 1293.e–1306.e. 10.1016/j.cell.2018.04.040 29775596 PMC6556895

[B4] Berson D. M. Dunn F. A. Takao M. (2002). Phototransduction by retinal ganglion cells that set the circadian clock. *Science* 295 1070–1073. 10.1126/science.1067262 11834835

[B5] Bray E. R. Yungher B. J. Levay K. Ribeiro M. Dvoryanchikov G. Ayupe A. C. (2019). Thrombospondin-1 Mediates Axon Regeneration in Retinal Ganglion Cells. *Neuron* 103 642–657.e7. 10.1016/j.neuron.2019.05.044 31255486 PMC6706310

[B6] Bumsted-O’Brien K. M. Hendrickson A. Harverkamp S. Ashery-Padan R. Schulte D. (2007). Expression of the homeodomain transcription factor Meis2 in the embryonic and postnatal retina. *J. Comp. Neurol.* 505 58–72. 10.1002/cne.21458 17729288

[B7] Chen C. Kiyama T. Weber N. Whitaker C. M. Pan P. Badea T. C. (2021). Characterization of Tbr2-expressing retinal ganglion cells. *J. Comp. Neurol.* 529 3513–3532. 10.1002/cne.25208 34245014 PMC8349894

[B8] Deneris E. S. Hobert O. (2014). Maintenance of postmitotic neuronal cell identity. *Nat. Neurosci.* 17 899–907. 10.1038/nn.3731 24929660 PMC4472461

[B9] Duan X. Qiao M. Bei F. Kim I. J. He Z. Sanes J. R. (2015). Subtype-Specific regeneration of retinal ganglion cells following axotomy: effects of osteopontin and mtor signaling. *Neuron* 85 1244–1256. 10.1016/j.neuron.2015.02.017 25754821 PMC4391013

[B10] Ecker J. L. Dumitrescu O. N. Wong K. Y. Alam N. M. Chen S. K. LeGates T. (2010). Melanopsin-expressing retinal ganglion-cell photoreceptors: cellular diversity and role in pattern vision. *Neuron* 67 49–60. 10.1016/j.neuron.2010.05.023 20624591 PMC2904318

[B11] Elsen G. E. Bedogni F. Hodge R. D. Bammler T. K. MacDonald J. W. Lindtner S. (2018). The epigenetic factor landscape of developing neocortex is regulated by transcription factors Pax6→ Tbr2→ Tbr1. *Front. Neurosci.* 12:571. 10.3389/fnins.2018.00571 30186101 PMC6113890

[B12] Feigl B. Mattes D. Thomas R. Zele A. J. (2011). Intrinsically photosensitive (melanopsin) retinal ganglion cell function in glaucoma. *Invest. Ophthalmol. Vis. Sci.* 52 4362–4367. 10.1167/iovs.10-7069 21498620

[B13] Gracitelli C. P. B. Duque-Chica G. L. Moura A. L. Roizenblatt M. Nagy B. V. de Melo G. R. (2016). Relationship between Daytime Sleepiness and Intrinsically Photosensitive Retinal Ganglion Cells in Glaucomatous Disease. *J. Ophthalmol.* 2016:5317371. 10.1155/2016/5317371 26955483 PMC4756205

[B14] Guillemot F. (2007). Spatial and temporal specification of neural fates by transcription factor codes. *Development* 134 3771–3780. 10.1242/dev.006379 17898002

[B15] Güler A. D. Ecker J. L. Lall G. S. Haq S. Altimus C. M. Liao H. W. (2008). Melanopsin cells are the principal conduits for rod-cone input to non-image-forming vision. *Nature* 453 102–105. 10.1038/nature06829 18432195 PMC2871301

[B16] Hatori M. Le H. Vollmers C. Keding S. R. Tanaka N. Schmedt C. (2008). Inducible ablation of melanopsin-expressing retinal ganglion cells reveals their central role in non-image forming visual responses. *PLoS One* 3:e2451. 10.1371/journal.pone.0002451 18545654 PMC2396502

[B17] Hattar S. Liao H. W. Takao M. Berson D. M. Yau K. W. Heller H. C. (2002). Melanopsin-Containing Retinal Ganglion Cells: architecture, Projections, and Intrinsic Photosensitivity. *Science* 295 1065–1070. 10.1126/science.1069609 11834834 PMC2885915

[B18] Hevner R. F. (2019). Intermediate progenitors and Tbr2 in cortical development. *J. Anat.* 235 616–625. 10.1111/joa.12939 30677129 PMC6656625

[B19] Legates T. A. Altimus C. M. Wang H. Lee H. K. Yang S. Zhao H. (2012). Aberrant light directly impairs mood and learning through melanopsin-expressing neurons. *Nature* 491 594–598. 10.1038/nature11673 23151476 PMC3549331

[B20] Li S. Woodfin M. Long S. S. Fuerst P. G. (2016). IPLaminator: an ImageJ plugin for automated binning and quantification of retinal lamination. *BMC Bioinform.* 17:36. 10.1186/s12859-016-0876-1 26772546 PMC4715356

[B21] Li S. Y. Yau S. Y. Chen B. Y. Tay D. K. Lee V. W. H. Pu M. L. (2008). Enhanced Survival of Melanopsin-expressing Retinal Ganglion Cells After Injury is Associated with the PI3 K / Akt Pathway. *Cell Mol. Neurobiol.* 28 1095–1107. 10.1007/s10571-008-9286-x 18512147 PMC11514987

[B22] Lin B. Masland R. H. (2006). Populations of Wide-Field Amacrine Cells in the Mouse Retina. *J. Comp. Neurol.* 499 797–809. 10.1002/cne.21126 17048228

[B23] Lyu P. Hoang T. Santiago C. P. Thomas E. D. Timms A. E. Appel H. (2021). Gene Regulatory Networks Controlling Temporal Patterning, Neurogenesis, and Cell Fate Specification in the Mammalian Retina. *SSRN Electron. J.* 37:109994. 10.2139/ssrn.3921283 PMC8642835 34788628

[B24] MacNeil M. A. Masland R. H. (1998). Extreme Diversity among Amacrine Cells: implications for Function. *Neuron* 20 971–982. 9620701 10.1016/s0896-6273(00)80478-x

[B25] Madisen L. Zwingman T. A. Sunkin S. M. Oh S. W. Zariwala H. A. Gu H. (2010). A robust and high-throughput Cre Reporting and characterization system for the whole mouse brain. *Nat. Neurosci.* 13 133–140. 10.1038/nn.2467 20023653 PMC2840225

[B26] Mao C. A. Kiyama T. Pan P. Furuta Y. Hadjantonakis A.-K. Klein W. H. (2008). Eomesodermin, a target gene of Pou4f2, is required for retinal ganglion cell and optic nerve development in the mouse. *Development* 135 271–280. 10.1080/1536710X.2013.870512.Behavioral 18077589 PMC2893890

[B27] Mao C. A. Li H. Zhang Z. Kiyama T. Panda S. Hattar S. (2014). T-box Transcription Regulator Tbr2 Is Essential for the Formation and Maintenance of Opn4/Melanopsin-Expressing Intrinsically Photosensitive Retinal Ganglion Cells. *J. Neurosci.* 34 13083–13095. 10.1523/JNEUROSCI.1027-14.2014 25253855 PMC4172803

[B28] Martersteck E. M. Hirokawa K. E. Evarts M. Bernard A. Duan X. Li Y. (2017). Diverse Central Projection Patterns of Retinal Ganglion Cells. *Cell Rep.* 18 2058–2072. 10.1016/j.celrep.2017.01.075 28228269 PMC5357325

[B29] Nadal-Nicolás F. M. Sobrado-Calvo P. Jimenez-Lopez M. Vidal-Sanz M. Agudo-Barriuso M. (2015). Long-Term Effect of Optic Nerve Axotomy on the Retinal Ganglion Cell Layer. *Invest. Ophthalmol. Vis. Sci.* 56 6095–6112. 10.1167/iovs.15-17195 26393669

[B30] Panda S. Sato T. K. Castrucci A. M. Rollag M. D. DeGrip W. J. Hogenesch J. B. (2002). Melanopsin (Opn4) requirement for normal light-induced circadian phase shifting. *Science* 298 2213–2216. 10.1126/science.1076848 12481141

[B31] Peng Y. R. Tran N. M. Krishnaswamy A. Kostadinov D. Martersteck E. M. Sanes J. R. (2017). Satb1 Regulates Contactin 5 to Pattern Dendrites of a Mammalian Retinal Ganglion Cell. *Neuron* 95 869.e–883.e. 10.1016/j.neuron.2017.07.019 28781169 PMC5575751

[B32] Pérez De Sevilla Müller L. Sargoy A. Rodriguez A. R. Brecha N. C. (2014). Melanopsin Ganglion Cells Are the Most Resistant Retinal Ganglion Cell Type to Axonal Injury in the Rat Retina. *PLoS One* 9:e93274. 10.1371/journal.pone.0093274 24671191 PMC3966869

[B33] Pérez De Sevilla Müller L. Shelley J. Weiler R. (2007). Displaced Amacrine Cells of the Mouse. *J. Comp. Neurol.* 505 177–189. 10.1002/cne 17853452

[B34] Pérez-Rico C. de la Villa P. Arribas-Gómez I. Blanco R. (2010). Evaluation of functional integrity of the retinohypothalamic tract in advanced glaucoma using multifocal electroretinography and light-induced melatonin suppression. *Exp. Eye Res.* 91 578–583. 10.1016/j.exer.2010.07.012 20692255

[B35] Pimeisl I. M. Tanriver Y. Daza R. A. Vauti F. Hevner R. F. Arnold H. H. (2013). Generation and characterization of a tamoxifen-inducible EomesCreER mouse line. *Genesis* 51 725–733. 10.1002/dvg.22417 23897762 PMC4112203

[B36] Provencio I. Cooper H. M. Foster R. G. Comp J. Lucas R. J. Res B. B. (2002a). Melanopsin (Opn4) Requirement for Normal Light-Induced Circadian Phase Shifting. *Science* 298 2213–2217. 12481141 10.1126/science.1076848

[B37] Provencio I. Rollag M. D. Castrucci A. M. (2002b). Photoreceptive net in the mammalian retina. *Nature* 415 493–494. 10.1038/415493a 11823848

[B38] Provencio I. Rodriguez I. R. Jiang G. Hayes W. P. Moreira E. F. Rollag M. D. (2000). A novel human opsin in the inner retina. *J. Neurosci.* 20 600–605. 10.1523/jneurosci.20-02-00600.2000 10632589 PMC6772411

[B39] Quattrochi L. E. Stabio M. E. Kim I. Ilardi M. C. Michelle Fogerson P. Leyrer M. L. (2018). The M6 cell: a small-field bistratified photosensitive retinal ganglion cell. *J. Comp. Neurol.* 527 297–311. 10.1002/cne.24556 30311650 PMC6594700

[B40] Rheaume B. A. Jereen A. Bolisetty M. Sajid M. S. Yang Y. Renna K. (2018). Single cell transcriptome profiling of retinal ganglion cells identifies cellular subtypes. *Nat. Commun.* 9:2759. 10.1038/s41467-018-05134-3 30018341 PMC6050223

[B41] Robinson G. A. Madison R. D. (2004). Axotomized mouse retinal ganglion cells containing melanopsin show enhanced survival, but not enhanced axon regrowth into a peripheral nerve graft. *Vis. Res.* 44 2667–2674. 10.1016/j.visres.2004.06.010 15358062

[B42] Rodriguez A. R. Perez De Sevilla Muller L. Brecha N. C. (2014). The RNA Binding Protein RBPMS is a Selective Marker of Ganglion Cells in the Mammalian Retina. *J. Comp. Neurol.* 522 1411–1443. 10.1002/cne.23521 24318667 PMC3959221

[B43] Ruby N. F. Brennan T. J. Xie X. Cao V. Franken P. Heller H. C. (2002). Role of melanopsin in circadian responses to light. *Science* 298 2211–2213. 10.1126/science.1076701 12481140

[B44] Sajgo S. Ghinia M. G. Brooks M. Kretschmer F. Chuang K. Hiriyanna S. (2017). Molecular codes for cell type specification in Brn3 retinal ganglion cells. *Proc. Natl. Acad. Sci.* 114 E3974–E3983. 10.1073/pnas.1618551114 28465430 PMC5441800

[B45] Sanes J. R. Masland R. H. (2015). The Types of Retinal Ganglion Cells: current Status and Implications for Neuronal Classification. *Annu. Rev. Neurosci.* 38 221–246. 10.1146/annurev-neuro-071714-034120 25897874

[B46] Schmidt T. M. Alam N. M. Chen S. Kofuji P. Li W. Prusky G. T. (2014). A role for melanopsin in alpha retinal ganglion cells and contrast detection. *Neuron* 82 781–788. 10.1016/j.neuron.2014.03.022.A 24853938 PMC4083763

[B47] Schmidt T. M. Kofuji P. (2009). Functional and morphological differences among intrinsically photosensitive retinal ganglion cells. *J. Neurosci.* 29 476–482. 10.1523/JNEUROSCI.4117-08.2009 19144848 PMC2752349

[B48] Seki T. Itoh H. Nakamachi T. Shioda S. (2008). Suppression of ganglion cell death by PACAP following optic nerve transection in the rat. *J. Mol. Neurosci.* 36 57–60. 10.1007/s12031-008-9091-5 18642101

[B49] Sessa A. Ciabatti E. Drechsel D. Massimino L. Colasante G. Giannelli S. (2017). The Tbr2 Molecular Network Controls Cortical Neuronal Differentiation Through Complementary Genetic and Epigenetic Pathways. *Cereb. Cortex* 27 3378–3396. 10.1093/cercor/bhw270 27600842

[B50] Sonoda T. Lee S. Birnbaumer L. Schmidt T. M. (2018). Melanopsin phototransduction is repurposed by ipRGC subtypes to shape the function of distinct visual circuits - preprint. *Neuron* 99 754–767.e4. 10.1016/j.neuron.2018.06.032 30017393 PMC6107377

[B51] Sonoda T. Li J. Y. Hayes N. W. Chan J. C. Okabe Y. Belin S. (2020). A noncanonical inhibitory circuit dampens behavioral sensitivity to light. *Science* 368 527–531. 32355031 10.1126/science.aay3152 PMC7512545

[B52] Srinivas S. Watanabe T. Lin C. S. William C. M. Tanabe Y. Jessell T. M. (2001). Cre reporter strains produced by targeted insertion of EYFP and ECFP into the ROSA26 locus. *BMC Dev. Biol.* 1:4. 10.1186/1471-213X-1-4 11299042 PMC31338

[B53] Stabio M. E. Sabbah S. Quattrochi L. E. Renna J. M. Briggman K. L. Berson D. M. (2018). The M5 cell: a color-opponent intrinsically photosensitive retinal ganglion cell. *Neuron* 97 150–163. 10.1016/j.neuron.2017.11.030 29249284 PMC5757626

[B54] Sweeney N. T. Tierney H. Feldheim D. A. (2014). Tbr2 Is Required to Generate a Neural Circuit Mediating the Pupillary Light Reflex. *J. Neurosci.* 34 5447–5453. 10.1523/JNEUROSCI.0035-14.2014 24741035 PMC3988404

[B55] Tran N. M. Shekhar K. Whitney I. E. Jacobi A. Benhar I. Hong G. (2019). Single-cell profiles of retinal neurons differing in resilience to injury reveal neuroprotective genes. *Neuron* 104 1039–1055.e12. 10.1016/j.neuron.2019.11.006 31784286 PMC6923571

[B56] Viney T. J. Balint K. Hillier D. Siegert S. Boldogkoi Z. Enquist L. W. (2007). Local Retinal Circuits of Melanopsin-Containing Ganglion Cells Identified by Transsynaptic Viral Tracing. *Curr. Biol.* 17 981–988. 10.1016/j.cub.2007.04.058 17524644

[B57] Wang H. Zhang Y. Ding J. Wang N. (2013). Changes in the Circadian Rhythm in Patients with Primary Glaucoma. *PLoS One* 8:e62841. 10.1371/journal.pone.0062841 23658653 PMC3639222

[B58] Yan W. Laboulaye M. A. Tran N. M. Whitney I. E. Benhar I. Sanes J. R. (2020). Mouse Retinal Cell Atlas: molecular Identification of Over Sixty Amacrine Cell Types. *J. Neurosci.* 40 5177–5195. 10.1523/jneurosci.0471-20.2020 32457074 PMC7329304

[B59] Ye D. Yang Y. Lu X. Xu Y. Shi Y. Chen H. (2019). Spatiotemporal Expression Changes of PACAP and Its Receptors in Retinal Ganglion Cells After Optic Nerve Crush. *J. Mol. Neurosci.* 68 465–474. 10.1007/s12031-018-1203-2 30415445

[B60] Zhu Y. Ju S. Chen E. Dai S. Li C. Morel P. (2010). T-bet and Eomesodermin Are Required for T Cell-Mediated Antitumor Immune Responses. *J. Immunol.* 185 3174–3183. 10.4049/jimmunol.1000749 20713880

