# Supplementary figures and images for: Adult Expression of Tbr2 Is Required for the Maintenance but Not Survival of Intrinsically Photosensitive Retinal Ganglion Cells
Source: Front Cell Neurosci. 2022 Mar 23;16:826590. doi: 10.3389/fncel.2022.826590 (PMC8983909; doi:10.3389/fncel.2022.826590)

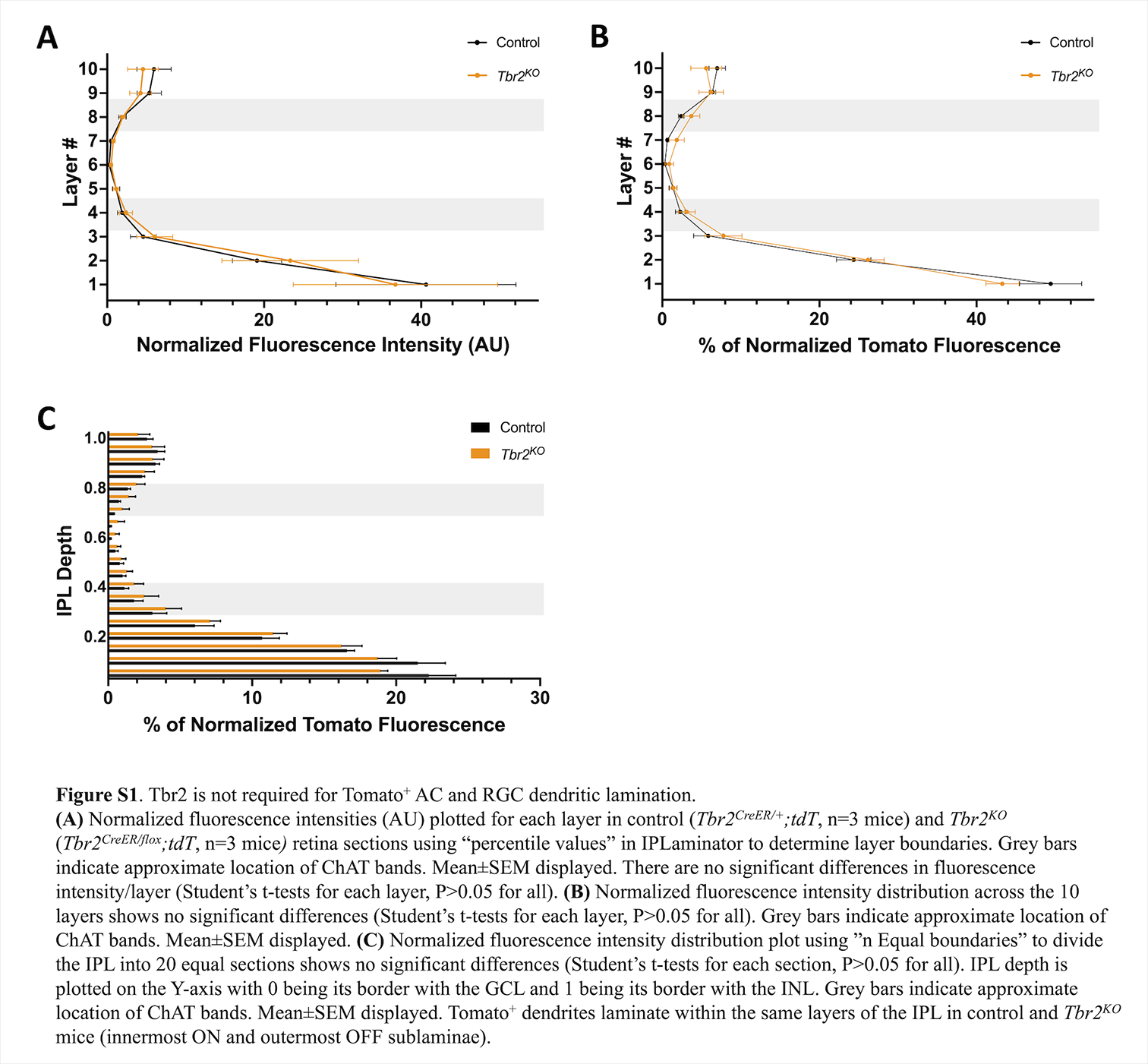

Supplement: Supplementary file 1 [file Image_1.TIFF]

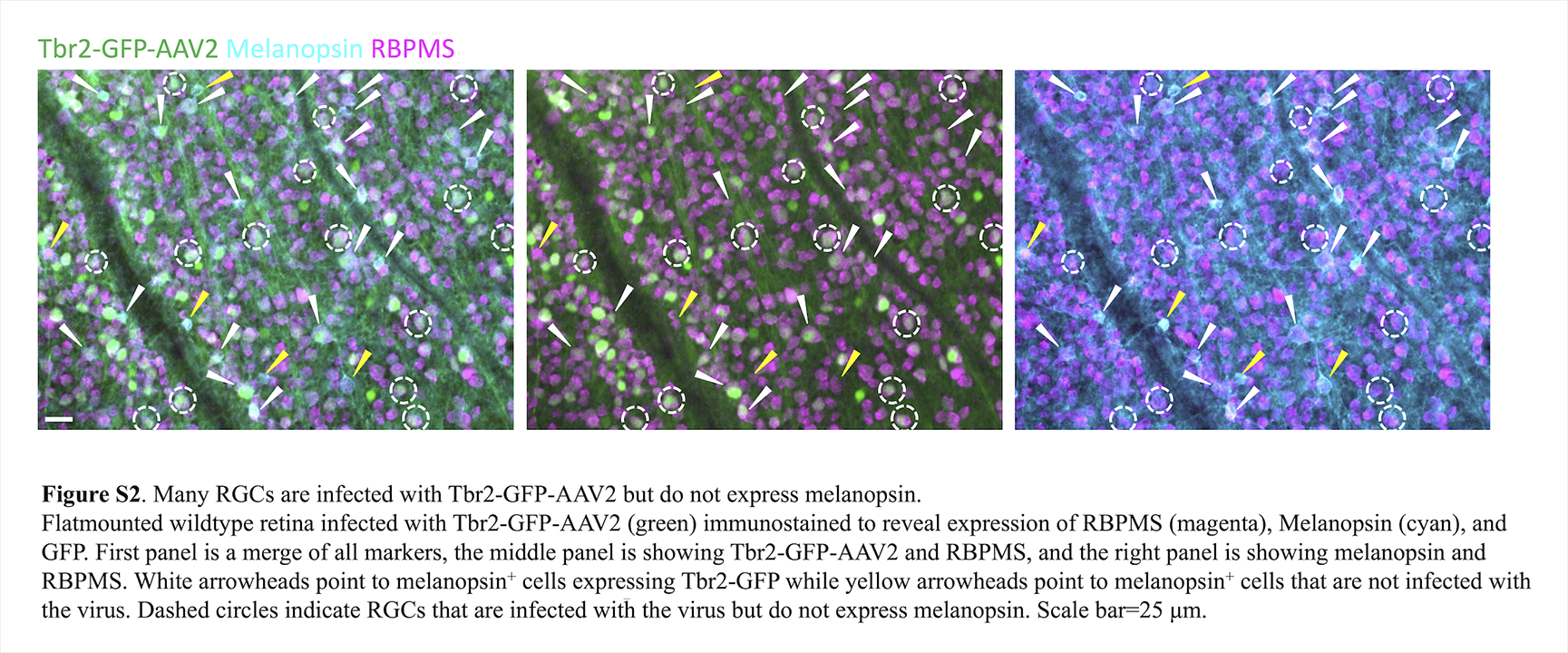

Supplement: Supplementary file 2 [file Image_2.TIFF]

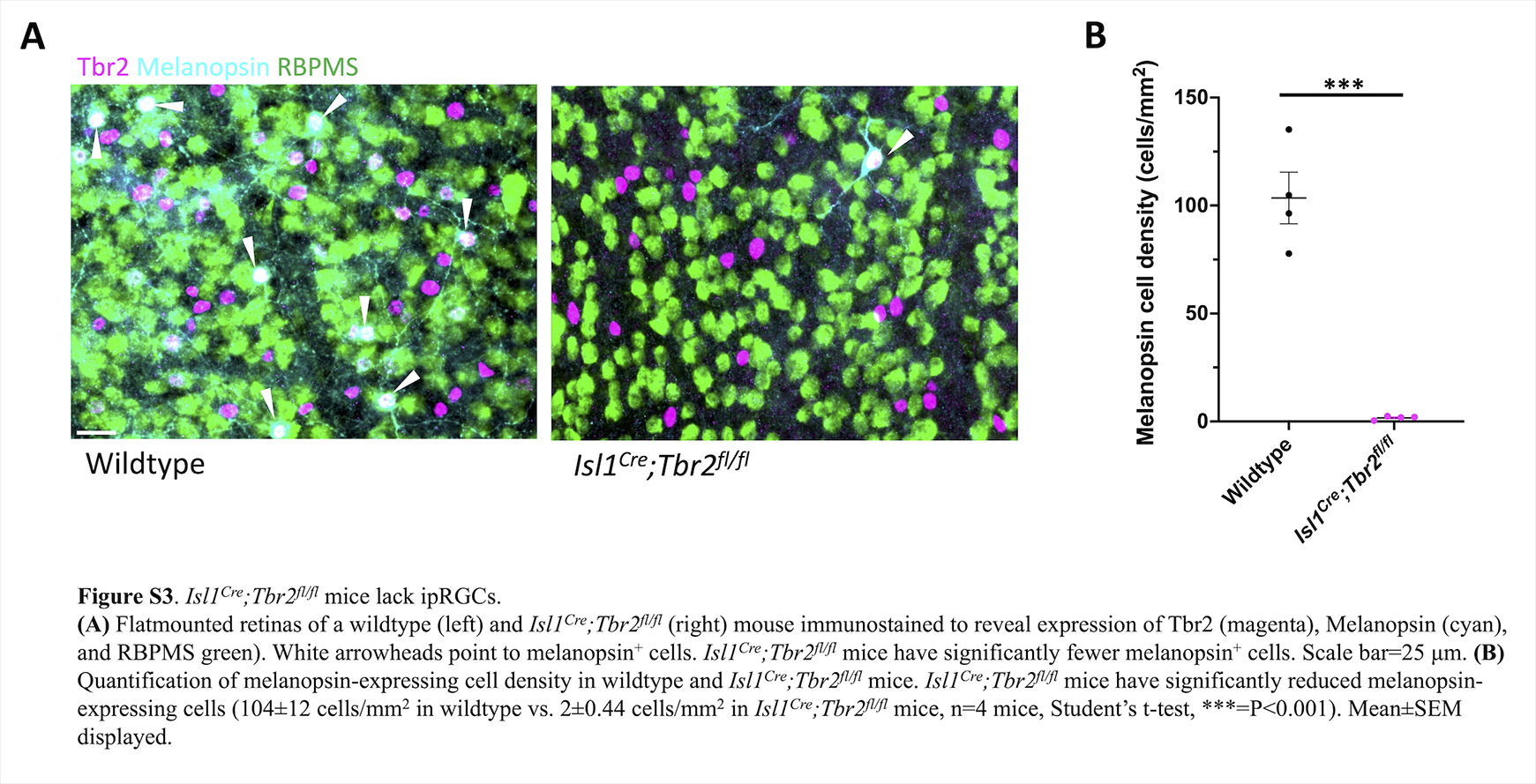

Supplement: Supplementary file 3 [file Image_3.TIFF]

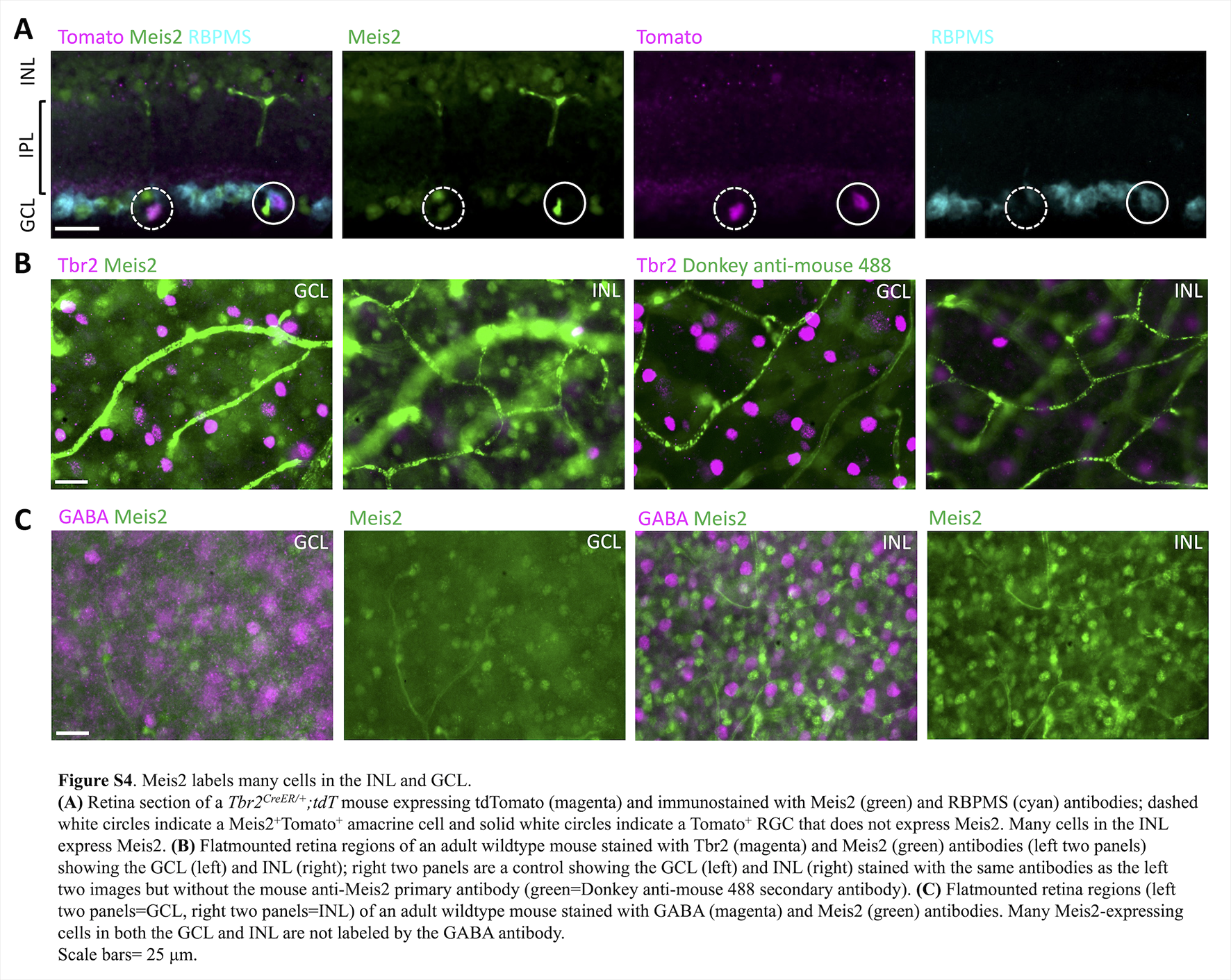

Supplement: Supplementary file 4 [file Image_4.TIFF]
